# Design of Flexible Conformal Beam-Scanning Leaky-Wave Antenna

**DOI:** 10.3390/mi17060657

**Published:** 2026-05-26

**Authors:** Jiahao Liu, Yiming Liu, Shuang Ma, Qilin Ren, Ya Fan, Zhongjie Wu, Xuebin Wang

**Affiliations:** 1 School of Electronic Information Engineering, Shenyang Aerospace University, Shenyang 110034, China; 2 Shandong Zhengzhong Information Technology Co., Ltd., Jinan 250098, China; liuym@sdas.org; 3 Air Force Early Warning Academy, Wuhan 430019, China; albert_fan028@foxmail.com; 4 College of Electronic Science, National University of Defense Technology, Changsha 410073, China; hit_wzj@163.com; 5 Communication Engineering Department, Aerospace Dongfanghong Satellite Co., Ltd., Beijing 100094, China

**Keywords:** conformal antenna, polyimide (PI), spoof surface plasmon polariton (SSPP), leaky-wave antennas (LWAs)

## Abstract

This paper presents a flexible conformal beam-scanning leaky-wave antenna (LWA) array based on a PI-ABS composite substrate and spoof surface plasmon polariton (SSPP) structure for Ku-band (12–18 GHz) applications. The proposed design features periodically symmetric gradient linear metallic stubs and interleaved tapered radiating patches to realize efficient SSPP slow-wave transmission, −1st spatial harmonic radiation, and open-stopband (OSB) suppression simultaneously. Benefiting from the flexible PI-ABS composite structure, the antenna maintains stable radiation performance under different curvatures, overcoming the mechanical instability and beam-scanning sensitivity of conventional flexible LWAs. The four-element conformal array achieves continuous beam scanning from −67° to 32° with a peak gain of 16.5 dBi and radiation efficiency above 58% across the entire band. Both simulation and measurement results validate that the proposed design integrates flexible conformality, wideband beam scanning, and high radiation efficiency, providing a novel solution for conformal wireless communication systems.

## 1. Introduction

In modern communication and detection systems [1], traditional rigid antennas can no longer meet practical demands. Compared to conventional rigid antennas, flexible antennas [2] can better conform to carrier surfaces, effectively reducing the antenna’s impact on mechanical structures. With the development of flexible [3] dielectric materials, antennas can better adapt to various types of curved substrates [4]. Furthermore, to enable optimal electromagnetic wave [5,6] reception under different motion states, research on the beam-scanning capability of antennas is of great significance. LWAs, with their advantages of controllable beam scanning, compact structure, and high radiation efficiency, can precisely match the coverage demands of dynamic signals in communication. Therefore, this paper address the limitations of traditional LWAs by developing a novel type of flexible conformal LWA [7] based on flexible dielectric substrates [8], forming an array configuration [9]. This approach balances flexibility and beam-scanning capability [10].

Typically, pre-fabricated rigid dielectric substrates can be used for conformal antennas. However, due to the rigidity of such materials, their curvature is fixed after fabrication, making them unable to adapt to surfaces with varying curvatures. This challenge can be overcome by employing flexible materials with stable performance and excellent bending characteristics. Materials such as polyethylene terephthalate (PET), polydimethylsiloxane (PDMS) [11], and PI are widely used as dielectric substrates for conformal antennas. By printing the antenna pattern onto these flexible dielectric substrates, they can conform well to curved substrates. PI is a high-performance organic polymer material featuring a low relative permittivity and excellent tensile properties, enabling it to conform well to curved substrates. Substrates with a lower permittivity can moderately increase the bandwidth of antennas while providing favorable impedance characteristics [12,13]. Acrylonitrile Butadiene Styrene (ABS), a common engineering plastic, offers excellent mechanical strength, formability, and cost-effectiveness. The combination of ABS and PI endows the antenna with both flexible characteristics and structural support stability.

Current research on LWAs primarily focuses on optimizing beam scanning [14] range, suppressing open-stopband interference, and mitigating electromagnetic interference [15]. However, relevant studies on conformal bending [16] remain scarce, and existing designs have obvious practical deficiencies. On one hand, existing flexible LWAs are predominantly based on a single layer of flexible substrate. While bendable, these lack sufficient mechanical strength, which leads to structural instability after long-term deformation and thus degrades the antenna performance [17,18]. This often results in discrepancies between the simulation and physical test results. On the other hand, traditional conformal [19] LWAs exhibit beam their beam-scanning capabilities are highly sensitive to curvature changes, frequently leading to issues such as sudden gain drops, unwanted electromagnetic interference [20], and scanning angle deviations.

To address these deficiencies, this paper proposes a novel continuous beam-scanning LWA [21,22] based on a PI-ABS composite flexible substrate integrated with an SSPP structure. First, the performance trends of a single LWA under different curvatures are simulated and verified, and the array antenna is physically tested on a substrate with a specific target curvature. Benefiting from the structural support of the PI-ABS composite substrate, the array can maintain good flexibility while conforming to the target curved surfaces, enabling extensive and stable beam scanning [23,24,25]. The proposed flexible conformal LWA array achieves continuous scanning from –67° rearward to 32° forward at 12–18 GHz, which outperforms most existing designs and is verified by physical tests.

The main novelties of this work are summarized as follows:

Material innovation: A PI-ABS composite flexible substrate is proposed, which combines the flexibility of PI and the mechanical support of ABS, solving the contradiction between antenna conformality and structural stability.

Structural innovation: Gradient SSPP gradient linear metallic stubs and interleaved tapered radiating patches are cooperatively designed, which realizes smooth mode transition, wideband radiation, and OSB suppression simultaneously.

Performance innovation: The conformal array achieves an ultra-wide beam-scanning range of −67° to 32° in 12–18 GHz, which outperforms most reported conformal LWAs.

## 2. Antenna Structure

The proposed conformal antenna array comprises four LWAs operating from 12 GHz to 18 GHz. Each LWA features identical structural parameters and element distribution. This uniformity contributes to the coupling between LWAs while also providing a structural foundation for subsequent conformal arrays.

**Figure 1:**
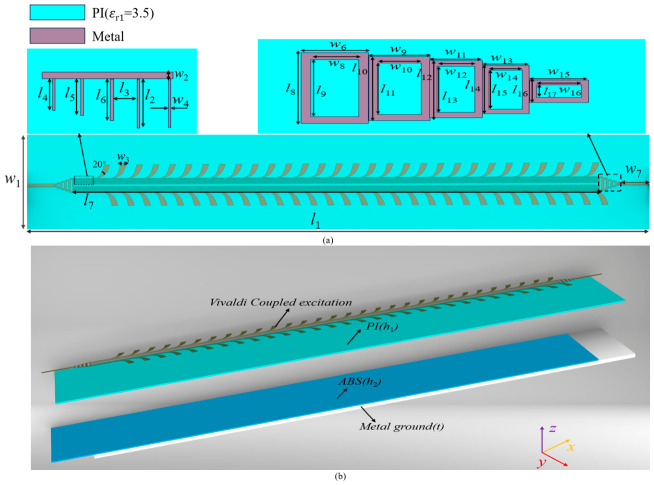
Structure of the proposed LWA. (**a**) Top view. (**b**) 3D hierarchical view.

Figure 1a illustrates the top view of the LWA, and its 3D hierarchical view is presented in Figure 1b. From top to bottom, the LWA consists of the antenna pattern printed on PI, the PI layer, the ABS layer, and the copper ground plane.

The PI layer serves as the dielectric substrate of the radiating structure and is the key medium enabling antenna flexibility. It has a relative permittivity of ε_r_ = 3.5, a loss tangent of tanδ = 0.002, and a thickness of h1 = 0.1 mm. These properties satisfy the bending requirements of the subsequent conformal array antenna while providing a low-loss platform for the metallic radiating pattern.

A 1 mm thick ABS layer is employed as a supporting structure to compensate for the insufficient mechanical rigidity of the PI layer. Its favorable mechanical strength and shock resistance ensure the structural integrity of the antenna.

The metallic pattern printed on the PI layer constitutes the radiation core of the antenna, which primarily consists of the feed structure, the SSPP transmission line, and a set of interleaved tapered radiating patches. Detailed parameters are shown in Table 1 below.

The conformal array comprises, from top to bottom, a metallic layer printed on a PI substrate, a PI dielectric layer, an ABS dielectric layer, and a copper ground plane. All layers share a uniform curvature radius of 100 mm. The conformal array consists of eight ports. Similarly to the single-LWA configuration, each port is equipped with tapered microstrip matching networks at both ends to provide standard 50 Ω coaxial interfaces for SMA connectors. These networks facilitate efficient mode conversion and ensure impedance matching with the SSPP transmission line.

**Figure 2:**
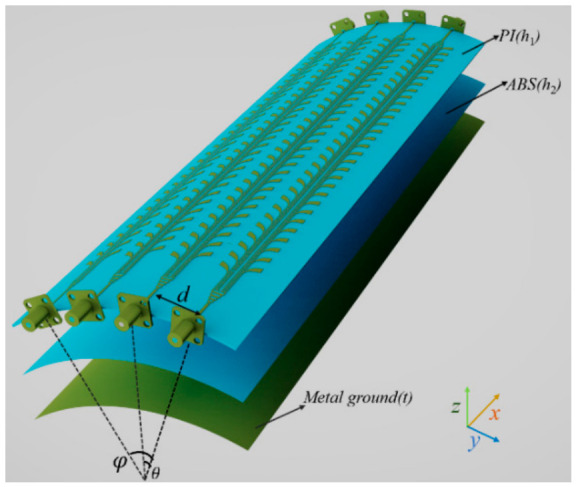
3D hierarchical view of the conformal antenna array (φ = 39°, θ = 13°, d = 5.2 mm).

The 3D hierarchical view of the conformal antenna array is shown in Figure 2. The spacing between two adjacent LWAs is set to 5.2 mm (d = 5.2 mm), corresponding to the half-wavelength spacing at the maximum frequency of 18 GHz.

## 3. Antenna Design Principles and Analysis

### 3.1. Single-Antenna Design: Leaky-Wave Radiation Mechanism Based on SSPP

SSPP is a type of artificial surface wave that simulates the transmission mode of surface plasmons through artificial periodic metallic structures. Since the SSPP on metallic branch units behaves as a slow wave and cannot radiate by itself, periodic perturbations must be introduced in accordance with the LWA theory for one-dimensional periodic structures. This converts the guided SSPP on metallic branch units into a Floquet wave, whose phase constant is given by $\beta_{n}=\beta_{0}+2\pi n/P$, where $\beta_{0}$ denotes the fundamental mode of the Floquet wave, n is the order of the spatial harmonic (n = 0, ±1, ±2, ±3, …), and P is the perturbation period. Periodic modulation can shift the original dispersion curve to excite an infinite number of spatial harmonics. Furthermore, through appropriate modulation, a specific harmonic branch can be tuned to the fast-wave region to satisfy the radiation condition of $\left|\beta_{-1}\right|<k_{0}$. The direction of the main beam can be calculated by the following formula:

(1) $$\theta_{n}=\arcsin\left(\frac{\beta_{-1}}{k_{0}}\right)=\arcsin\left(\frac{\beta_{0}}{k_{0}}-\frac{\lambda_{0}}{P}\right)$$

Conventional SSPP structures typically employ uniformly periodic metallic teeth. However, such configurations suffer from discontinuous mode excitation. When directly interfaced with a microstrip line that supports a quasi-transverse electromagnetic (quasi-TEM) mode, the abrupt mode transition leads to significant energy reflection, thereby hindering both slow-wave propagation and leaky-wave radiation. To address this issue and facilitate the SSPP mode, a tapered transition consisting of gradually varying linear metallic stubs is adopted. A fixed-step linear gradient is utilized here: the front stubs grow stepwise by 0.05 mm until the fourth stub, after which the stub length remains unchanged. The rear stubs are symmetrically arranged and reduce sequentially by 0.05 mm. The stub length progressively increases from the microstrip feed end toward the SSPP section, enabling a smooth impedance transition between the quasi-TEM mode and the SSPP mode.

**Figure 3:**
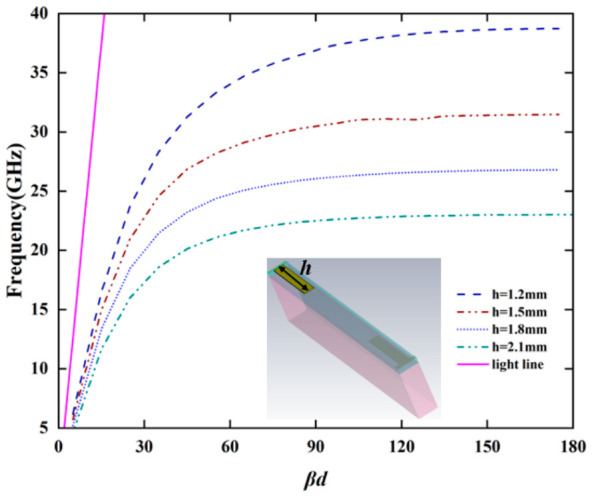
SSPP transmission dispersion curve.

Figure 3 presents the dispersion curve of the LWA waveguide. The asymptotic frequency decreases as the groove depth h increases, providing a theoretical basis for the tapered design of SSPP stubs. Moreover, the dispersion curves of the proposed unit structure lie below the light line, confirming the slow-wave characteristics inherent to the SSPP waveguide.

The dispersion characteristics of the proposed SSPP unit cell are computed and shown in Figure 3. As expected, the dispersion curve lies entirely below the light line, confirming the slow-wave nature. The asymptotic frequency decreases with increasing stub length h, which is the physical basis for the frequency-dependent propagation constant.

As analyzed above, the metallic branch unit functions as a slow-wave structure. To radiate the guided electromagnetic waves into free space, periodic perturbations must be introduced. Periodically symmetric metallic stubs are employed as the periodic perturbation unit of the LWA. The symmetric structure stabilizes the SSPP transmission and systematically modulates the guided slow wave, which accurately excites the −1st spatial harmonic and avoids unwanted higher-order harmonic interference.

### 3.2. Single-Antenna Design: Radiation Analysis of Tapered Radiating Patches

**Figure 4:**
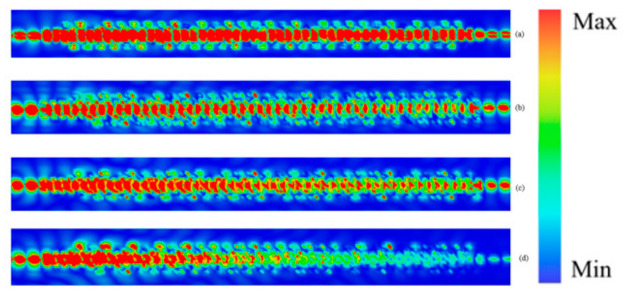
Simulated electric field distribution at 12 GHz under different curvatures. (**a**) Normal operating conditions; (**b**) curvature radius of 100 mm; (**c**) curvature radius of 60 mm; (**d**) curvature radius of 40 mm.

However, in the sidelobe directions, spatial harmonics—such as β0 and −β-2, β1 and −β-3, β2 and −β-5—interfere with each other and superimpose in pairs, leading to the formation of standing waves. This results in abrupt impedance variations, causing significant reflections and severe degradation of the radiation pattern, a phenomenon known as the open-stopband (OSB) effect. To suppress the OSB, interleaved tapered radiating patches are integrated on both sides of the SSPP stub array. The interleaved placement breaks the exact periodicity of the stubs, while the tapered radiating patches profile further reduces the effective periodic modulation strength for the undesired harmonics. As a result, the condition for standing-wave formation is destroyed. This mechanism is verified by the electric field distribution shown in Figure 4, where the fields are gradually radiated along the structure without strong reflections. Moreover, the radiating patches themselves act as progressive radiators: they couple the SSPP guided wave and convert it into a radiating wave over a wide frequency range, thereby enhancing the operating bandwidth.

From the perspective of radiation mechanism, the tapered radiating patches and SSPP stubs form a synergistic radiation structure:

- The SSPP stubs provide tight field confinement and stable slow-wave transmission;
- The interleaved radiating patches couple the slow-wave energy from the SSPP structure and radiate it gradually into free space;
- The tapered impedance of radiating patches broadens the operating bandwidth and improves impedance matching.

To verify whether the tapered radiating patches still cooperate with the SSPP metallic stubs for wave transmission and radiation under different curvatures, the electric field characteristics of the proposed antenna unit are analyzed at 12 GHz with curvature radii of 100 mm, 60 mm, and 40 mm, together with the normal case. 

Figure 4a–d present the simulated total electric field distribution at 12 GHz under different curvatures. It can be seen that the SSPP mode is effectively excited and propagates along the SSPP waveguide structure. When tapered radiation patches are placed around the waveguide, the SSPP mode is easily perturbed and transmitted to the patches for the radiation. As the electromagnetic wave energy is progressively radiated through the tapered patches, the electric field intensity along the antenna structure decreases accordingly.

As shown in Figure 4, for the planar antenna, the surface electric field exhibits a uniform and regular periodic distribution along the antenna length, indicating a stable traveling-wave propagation state and consistent energy leakage characteristics. When the antenna is bent to a radius of 100 mm, the overall electric field distribution remains essentially consistent with that of the planar case. The periodic pattern of the electric field is still clearly observable, with no significant distortion or substantial attenuation. This demonstrates that moderate bending has virtually no impact on electromagnetic wave propagation. As the bending radius is reduced to 60 mm, slight variations in the amplitude and distribution of the electric field can be observed. However, the main periodic characteristics of the traveling wave are still preserved, and the overall energy leakage behavior of the antenna remains within a controllable range. At a bending radius of 40 mm, although slight distortion and attenuation of the electric field occur, the antenna still maintains relatively clear traveling-wave propagation characteristics, with no destructive resonance or field collapse observed.

These results not only confirm that the tapered patches cooperate with the SSPP metallic stubs to achieve wave transmission and radiation, but also demonstrate that this cooperative mechanism remains stable under different curvatures. Overall, the proposed antenna element maintains relatively stable transmission and radiation performance under different curvatures, which lays a foundation for further research on its radiation characteristics under bending.

### 3.3. Single-Antenna Design: Radiation Analysis of LWA Under Different Curvatures

The radiation characteristics of a single LWA element under diverse curvatures are simulated to establish a foundation for the subsequent conformal antenna array design. The corresponding one-dimensional radiation pattern is illustrated in the following section.

**Figure 5:**
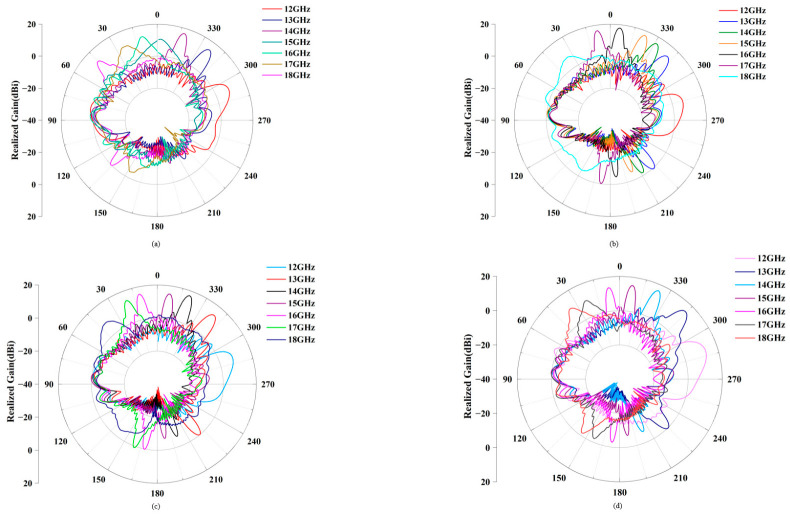
(**a**) Simulated H-plane radiation patterns of the LWA over the 12–18 GHz band under normal operating conditions. (**b**) Simulated H-plane radiation patterns of conformal LWA (radius = 100 mm) over 12–18 GHz. (**c**) Simulated H-plane radiation patterns of conformal LWA (radius = 60 mm) over 12–18 GHz. (**d**) Simulated H-plane radiation patterns of conformal LWA (radius = 40 mm) over 12–18 GHz.

Figure 5a–d present the corresponding H-plane radiation patterns. Within the 12–18 GHz band, the radiating element achieves continuous beam scanning for the LWA directivity while maintaining stable scanning performance under different curvatures. This stability provides a foundation for consistent radiation patterns in subsequent array implementations.

Through the simulation analysis of radiation patterns, the stability of the single flexible LWA is verified, thereby establishing a reliable basis for the design of conformal arrays.

### 3.4. Principles of Conformal Array Design: Performance Enhancement Based on Beamforming

Beamforming is a core technique in which an antenna array enhances radiation by superimposing radiation fields from specific directions through the control of the amplitude and phase of individual antenna elements. Based on this technique, this paper combines the characteristics of SSPP elements with a flexible conformal structure to design the directional radiation performance of a four-antenna conformal array. The spacing between adjacent antennas is designed based on the sidelobe suppression condition of beamforming, d<λ/2 (the half-wavelength spacing at the maximum frequency of 18 GHz). This helps prevent the appearance of additional lobes within the target frequency band.

The conformal array in this paper employs a half-wavelength equidistant arrangement. The spacing between adjacent antennas is set to λ/2 (5.2 mm) to ensure that the radiation energy is concentrated in the main lobe. Benefiting from the flexibility of the PI-ABS composite substrate, when the array is attached to a curved carrier, the four LWAs can conform to different positions on the carrier surface with the deformation of the substrate. This changes the coordinates of each LWA in its relative coordinate system. Consequently, the radiated harmonics from each LWA generate phase differences at specific spatial positions due to the different propagation paths. These phase differences form an adaptive adjustment of the radiation phase based on the curvature of the carrier. Thus, the main beam direction can be maintained in the orientation adapted to the curved surface, while the spacing between the antennas is still constrained by the half-wavelength requirement. This provides a stable spatial array structure for beamforming in conformal applications.

**Figure 6:**
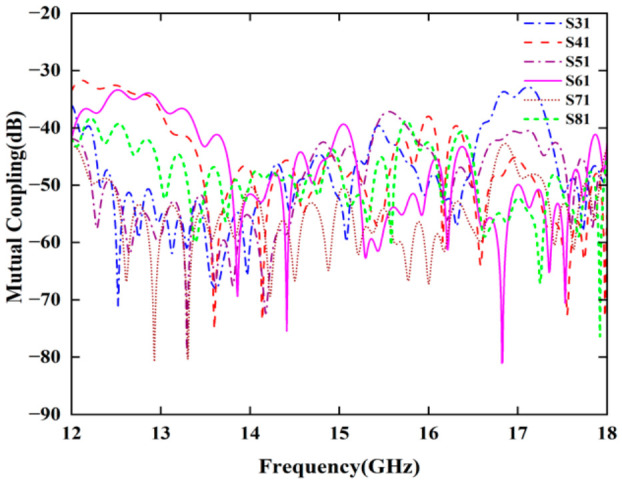
Channel coupling coefficient.

Figure 6 presents the channel coupling coefficient between antennas in the flexible conformal array. Within the operating frequency band, the mutual coupling is below −30 dB, indicating that the coupling has a negligible effect on the radiation characteristics of the conformal array under the adopted spacing arrangement.

### 3.5. Comparative Analysis of the Array Port S11 and the Antenna Unit S11

Given that coupling occurs between the individual elements of the array, the S11 parameters of the array will inevitably differ from those of a single LWA element. With this in mind, we selected one LWA element for comparison and performed an S11 port analysis on it relative to the array.

**Figure 7:**
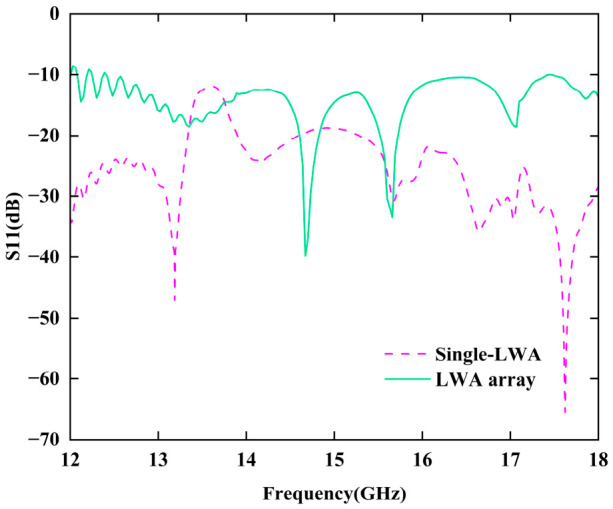
Comparison of S11 values between the array and one of the LWA units.

As shown in the Figure 7, the average loss of a single LWA is lower than that of an array, with S11 as low as −60 dB at certain frequencies.

This is primarily because, for a single antenna element, the input impedance is determined solely by its structural parameters and radiation characteristics. However, when multiple antenna elements are arranged in an array, the electromagnetic field radiated by one element couples into the ports of adjacent elements, introducing additional reactive and resistive components into the input impedance. These coupled fields alter the original impedance matching conditions, leading to increased reflection at the feed port and, ultimately, higher S11parameter values compared to a single antenna element.

### 3.6. Analysis of the Influence of Different Carriers on Conformal Arrays

As conformal antennas are typically integrated with substrates of various materials, their performance was evaluated on both metallic and non-metallic substrates. The center frequency of 15 GHz, located within the operating band, was selected for the 3D radiation pattern analysis.

**Figure 8:**
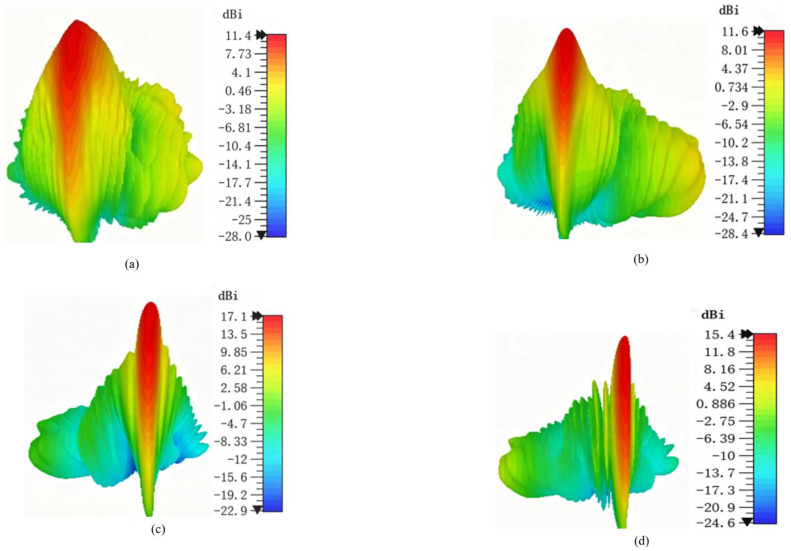
Three-dimensional radiation patterns of conformal arrays at 15 GHz with FR4 non-metallic) carriers of different thicknesses. (**a**) Carrier thickness of 0.018 mm. (**b**) Carrier thickness of 0.518 mm. (**c**) Carrier thickness of 1.018 mm. (**d**) Carrier thickness of 1.518 mm.

The three-dimensional radiation patterns of the conformal antenna array operating at 15 GHz with non-metallic FR4 carriers of varying thicknesses (0.018 mm, 0.518 mm, 1.018 mm, and 1.518 mm) are illustrated in Figure 8a–d.

A distinct trend is observed: as the carrier thickness increases from 0.018 mm to 1.018 mm, the peak realized gain exhibits a gradual upward tendency. Specifically, the array incorporating a 0.018 mm thick carrier achieves the lowest peak gain of 11.4 dBi, whereas the 0.518 mm thick counterpart demonstrates a modest improvement to 11.6 dBi. Optimal performance is attained with the 2.5 mm thick carrier, which delivers the highest peak gain of 1.018 dBi, coupled with a well-defined main beam and substantially suppressed sidelobes.

Nevertheless, a further increase in thickness to 1.518 mm results in a moderate attenuation in performance, with the peak gain decreasing to 15.4 dBi. This reduction can be attributed to elevated dielectric loss in relatively thick carriers, effects that not only slightly compromise radiation efficiency but also introduce minor undesirable spurious lobes without significant distortion of the radiation pattern.

**Figure 9:**
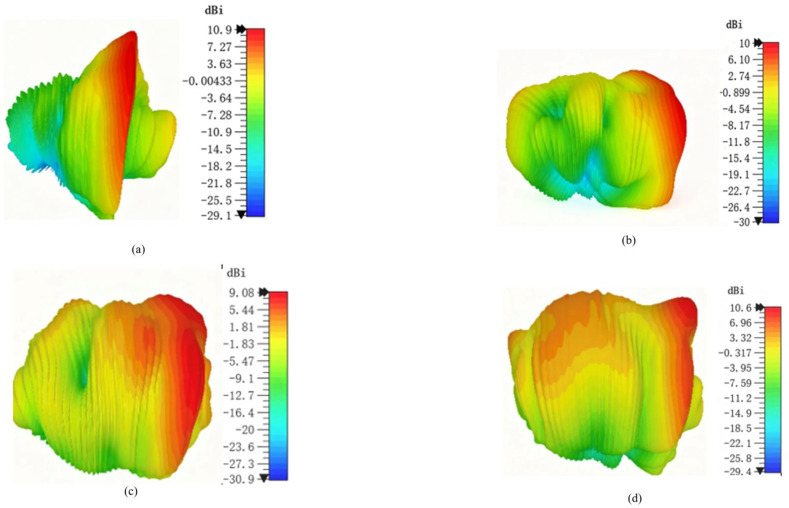
Three-dimensional radiation patterns of conformal arrays at 15 GHz with copper (metallic) carriers of different thicknesses. (**a**) Carrier thickness of 0.018 mm. (**b**) Carrier thickness of 0.518 mm. (**c**) Carrier thickness of 1.018 mm. (**d**) Carrier thickness of 1.518 mm.

The 3D far-field radiation patterns of the conformal leaky-wave antenna array mounted on copper carriers with different thicknesses (0.018 mm, 0.518 mm, 1.018 mm, and 1.518 mm) at 15 GHz are presented in Figure 9a–d. A clear trend of performance degradation with increasing carrier thickness is observed.

For the 0.018 mm thick copper carrier, the array exhibits favorable radiation characteristics, with a well-formed, highly directional main beam. This ultra-thin copper layer effectively acts as a ground plane, reflecting backward-radiated energy into the forward hemisphere while introducing minimal parasitic effects. Consequently, the radiation pattern features a well-defined main lobe, relatively low sidelobe levels, and strong boresight directivity.

As the copper carrier thickness increases to 0.518 mm, the radiation pattern begins to degrade. The main beam loses its sharpness and becomes broader, accompanied by increased undesired radiation in off-boresight directions. This indicates that the thicker metallic carrier introduces stronger mutual coupling between array elements and more pronounced surface wave effects, which degrade beam collimation and elevate sidelobe levels.

Further performance degradation is observed for the 1.018 mm and 1.518 mm cases, where the peak realized gains are 9.08 dBi and 10.6 dBi, respectively. Both patterns exhibit severely distorted main beams, significantly elevated sidelobes, and a clear reduction in directivity. Although the 1.518 mm configuration shows a slight recovery in peak gain, the beam shape remains highly irregular, with radiated energy scattered across multiple directions, failing to form a well-focused main lobe.

Therefore, for both metallic and non-metallic carriers, there exists a critical thickness beyond which the radiation performance of the conformal array degrades significantly. Furthermore, the performance of the array is more sensitive to variations in the thickness of the metallic carrier than to those of the non-metallic counterpart.

## 4. Physical Processing and Testing

**Figure 10:**
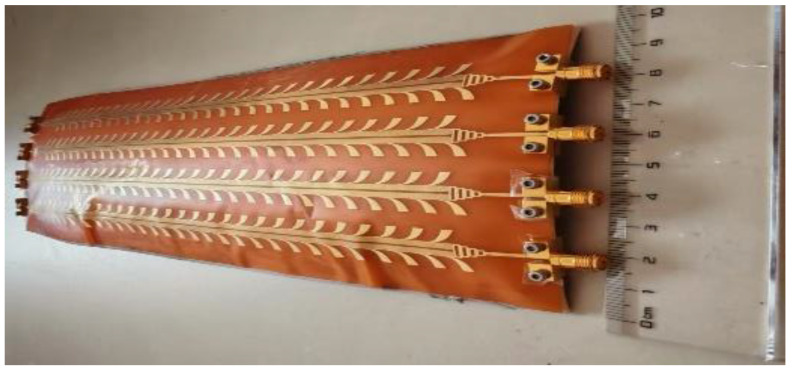
Physical prototype of the proposed flexible conformal array.

To further validate the feasibility of the conformal array, the proposed conformal array was fabricated and tested (Figure 10).

The array radiation layer employs a flexible PI film with single-sided patterned copper plating as its core dielectric substrate. Fabricated using a double-sided copper-clad PI board as the base material, the manufacturing process includes cutting, laser drilling, dry film patterning, electrochemical acid etching, gold plating, and laser contour cutting. Corresponding quality inspections are implemented throughout each step, ultimately yielding the target PI substrate.

ABS is a common engineering plastic, and a fan-shaped conformal solid with a radius of 100 mm is fabricated by 3D printing technology as the support structure beneath the PI film. Specialized tools are used to perform fine trimming on both sides of the ABS support structure, to ensure that all critical dimensional tolerances are controlled within 0.05 mm. A copper foil structure is adopted for the metallic ground layer. A pressure-sensitive adhesive copper foil with a thickness of 0.017 mm is selected. After removing the protective film, the copper foil is precisely aligned and attached to the ABS substrate, and covers the entire rear surface of the array to ensure the unidirectional radiation of the array. Finally, the assembly of the PI film and ABS support structure is completed. The core challenge is to achieve bubble-free bonding between the PI film and the ABS substrate, as well as conformal consistency of the three-layer structure. An ultra-thin double-sided adhesive tape matching the size of the PI film is selected. One release liner of the tape is removed, and the tape is precisely aligned with the back surface of the PI film. Light pressure is applied to the center of the tape with a roller, and then the tape is rolled outward gradually from the center. A pressure of 0.3–0.5 MPa is maintained during the rolling process to ensure complete adhesion without air bubbles. The excess tape is trimmed along the edges of the PI film with a blade to achieve flush alignment. The ABS substrate is fixed in a custom-made positioning fixture, with the conformal surface facing upward and kept stable. The remaining release liner of the double-sided adhesive tape on the PI film is removed. First, the center of the PI film is aligned with that of the ABS substrate, and light pressure is applied. Then, pressure is applied radially outward from the center to ensure that all air is completely expelled from the bonding interface. For the convex areas of the conformal surface, pressure is applied gradually along the contour to prevent the PI film from wrinkling or stretching.

It should be noted that during the fabrication of the PI and ABS components, four pairs of screw holes with a diameter of 2.1 mm must be pre-drilled on both the front and back surfaces to facilitate subsequent screw fastening. Solderless SMA connectors are employed for assembly and experimental testing.

**Figure 11:**
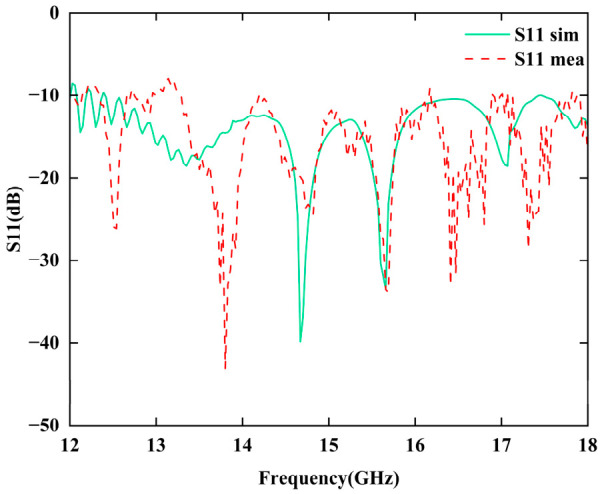
Measured and simulated |S11| of conformal array.

Figure 11 shows the measured |S11| parameters. The results demonstrate that the conformal array achieves good impedance matching across the 12–18 GHz frequency band. The measured results exhibit an overall frequency deviation of approximately 0.6 GHz from the simulations, which is mainly caused by manual assembly errors of the SMA connector, unstable contact between the PI film and ABS substrate that fails to achieve ideal tight adhesion, and the uncertainties of the actual test environment. Furthermore, the minimum measured |S11| value approaches –50 dB, indicating that the reflection coefficient of the conformal array is significantly reduced, with most of the input energy radiated outward through the gaps in the array.

**Figure 12:**
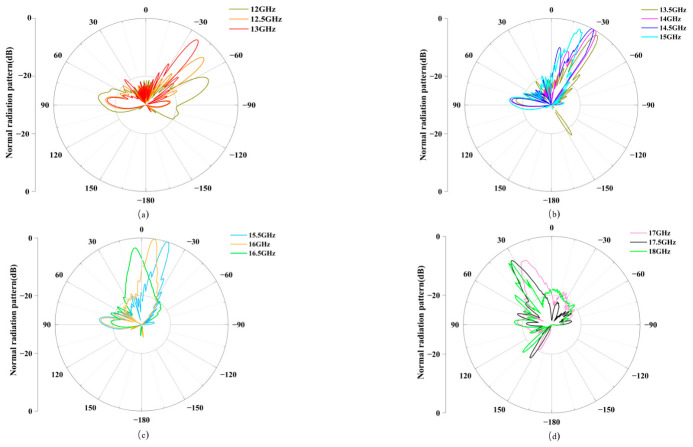
(**a**–**d**) Simulated H-plane radiation patterns of the conformal array.

Figure 12a–d show the normalized H-plane radiation patterns of the array. The simulation results show that the actual beam-scanning range of the proposed flexible LWA array is from –67° to 32°. The patterns exhibit a monotonic evolution of the main lobe with increasing frequency, with no significant beam splitting or pattern distortion observed across the entire band. The sidelobe levels remain consistently below −10 dB relative to the main beam, ensuring a high degree of radiation directivity. This stable frequency-scanning behavior verifies the effectiveness of the conformal design in maintaining the desired leaky-wave radiation characteristics, enabling broad angular coverage for beam-scanning applications.

**Figure 13:**
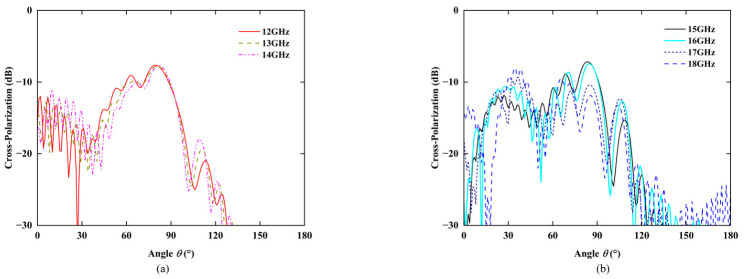
(**a**,**b**) Simulation results of cross-polarization of conformal arrays.

As depicted in the cross-polarization characteristic curves, the peak cross-polarization magnitude within the main beam exhibits frequency-dependent fluctuations across the operating bandwidth of 12–18 GHz. Within the 12–15 GHz band, the peak main-beam cross-polarization is marginally higher than −10 dB and approximates this value, achieving a polarization isolation level close to 10 dB. As the operating frequency increases further, this parameter gradually declines to −16 dB at 18 GHz (Figure 13).

Such frequency-dependent variation originates from the conformal installation configuration and intrinsic electromagnetic properties of the designed SSPP leaky-wave antenna. Bending deformation of the flexible substrate disrupts the inherent field distribution symmetry of the antenna structure. Combined with strong radiative energy leakage and structural disturbance induced by conformal curvature, the orthogonal polarization component within the main beam is correspondingly enhanced. Furthermore, the locally arranged interleaved tapered radiating patches structure, which is adopted to suppress open-stopband effects, introduces subtle structural asymmetry, which further raises the main-beam cross-polarization level.

Overall, the cross-polarization performance is typical for flexible conformal leaky-wave antennas. This performance is acceptable for practical conformal communication applications that do not require high polarization diversity, and it does not compromise the antenna’s core beam-scanning functionality.

**Figure 14:**
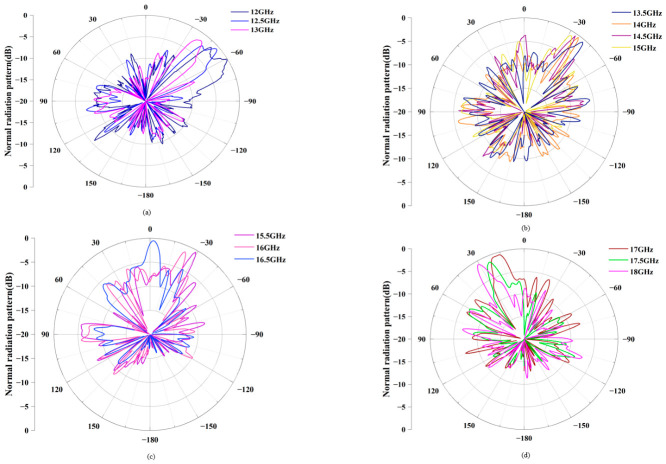
(**a**–**d**) Measured H-plane radiation patterns of the conformal array.

Figure 14a–d show the normalized H-plane radiation patterns of the array. Measurements show that the actual beam-scanning range of the proposed flexible LWA array is from –67° to 32°, with obvious directivity, which validates the beam-scanning performance of the flexible conformal array.

By comparing simulated and measured H-plane radiation patterns, the overall frequency-scanning beam steering trend, angular coverage range and main lobe evolution law of the experimental results are in good agreement with the simulation predictions. The measured maximum backward scan angle −67° and maximum forward scan angle +32° are highly consistent with the simulated results, which verifies the correctness of the conformal LWA array design and simulation modeling.

In contrast, the measured peak sidelobe levels at individual frequency points are obviously higher than simulated ideal values. The simulated patterns exhibit low, stable sidelobe suppression across the whole band, while partial measured frequency points suffer from degraded sidelobe characteristics. As analyzed above, this deviation mainly originates from anechoic chamber environmental interference and unstable SMA fixture contact during far-field testing rather than structural design defects of the antenna itself. In general, the measured radiation performance effectively verifies the frequency scanning performance of the proposed conformal leaky-wave antenna array.

**Figure 15:**
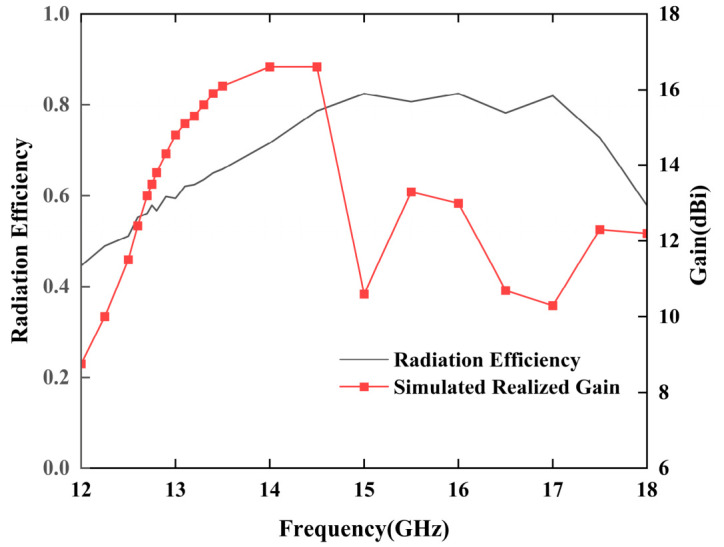
Conformal array simulation of simulated realized gain and radiation efficiency.

The simulated radiation efficiency and realized gain of the proposed conformal leaky-wave antenna array are presented in Figure 15, spanning the entire operating bandwidth of 12–18 GHz.

As shown by the black curve, the radiation efficiency exhibits a steadily increasing trend from approximately 45% at 12 GHz, peaking above 80% across the mid-to-high frequency band (14–17 GHz), with a maximum value of 83% achieved at 15 GHz. Even at the upper edge of the band (18 GHz), the efficiency remains above 58%, demonstrating that the proposed structure maintains excellent radiation characteristics over the full operating range, with minimal spurious wave leakage and good energy radiation capability.

The red square markers represent the simulated realized gain. At lower frequencies (12–14.5 GHz), the gain rises rapidly from 8.6 dBi to a peak of 16.5 dBi at 14–14.5 GHz, which corresponds to the optimal radiation efficiency region. A slight drop is observed at 15 GHz, which can be attributed to the transition effect of the leaky-wave radiation mode, while the gain remains above 10 dBi across the entire band. Throughout the 15–18 GHz range, the gain fluctuates within a range of 10–13 dBi, maintaining a stable and high level.

It should be emphasized that the radiation efficiency varies across the operating band, reaching approximately 45% at 12 GHz, peaking at 83% at 15 GHz, and remaining above 58% at 18 GHz. At the lower band edge (12 GHz), the SSPP mode exhibits strong slow-wave confinement and a low leakage rate of the −1st spatial harmonic, resulting in insufficient radiated energy and a relatively low radiation efficiency of about 45%. At the upper band edge (18 GHz), the leakage rate becomes excessively high, making the antenna over-leaky and shortening the effective radiation aperture, which reduces the efficiency to approximately 58%. The peak efficiency of 83% near 15 GHz corresponds to an optimal leakage rate that balances the under-leaky and over-leaky regimes for the given antenna length. Above 15 GHz, the realized gain exhibits a moderate reduction, while the radiation efficiency remains stable between 0.75 and 0.82 in this frequency range. Combined with the excellent |S11| performance (<−10 dB) across the entire operating band, this confirms that the gain reduction is not caused by poor impedance matching or low radiation efficiency. Instead, it arises primarily from the rapidly increasing leakage rate of the −1st-order spatial harmonic associated with the SSPP mode at high frequencies. This effect shortens the antenna’s effective radiation aperture and reduces the directivity of the main beam. Despite this reduction, the realized gain remains above 10 dBi from 15 to 18 GHz, and the radiation patterns remain stable without distortion, fully meeting the requirements of practical conformal communication applications.

Overall, the array achieves a high radiation efficiency above 58% and a realized gain above 8.6 dBi across the entire 12–18 GHz band, with peak values reaching 83% and 16.5 dBi, respectively. These results confirm that the proposed conformal leaky-wave antenna array offers high radiation efficiency and stable gain performance over the full Ku-band, validating its suitability for high-efficiency wideband communication applications.

**Figure 16:**
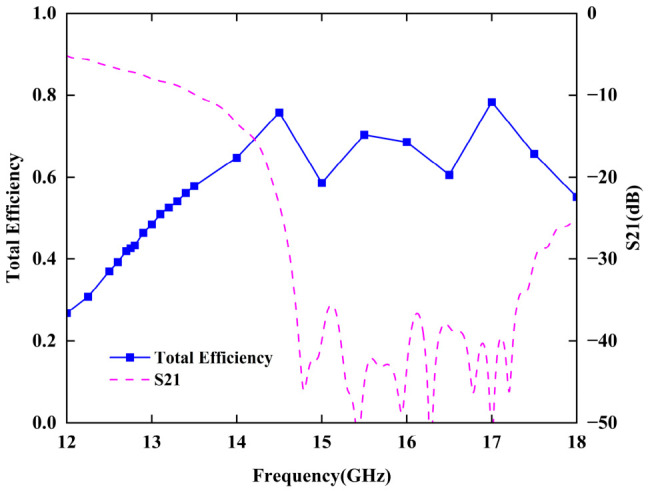
Conformal array simulation of total efficiency and S21 parameters.

Figure 16 presents the simulated total efficiency and transmission coefficient S21 of the conformal leaky-wave antenna array across the operating frequency band of 12–18 GHz. A clear correlation can be observed between these two metrics.

At lower frequencies (12–14 GHz), the array exhibits a total efficiency that gradually increases from approximately 0.25 to 0.75. Meanwhile, the magnitude of S21 remains close to 0 dB, indicating strong mutual coupling between adjacent elements. This behavior indicates that, within this frequency range, guided-wave energy is primarily transmitted between elements, with relatively less energy radiated into free space compared to other frequency bands.

As the frequency increases beyond 14 GHz, the relationship between the two parameters becomes more complex. The total efficiency reaches its peak and then fluctuates between approximately 0.5 and 0.8, while the S21 magnitude drops sharply to below −20 dB, with several deep notches extending below −40 dB. This significant reduction in S21 indicates that inter-element coupling is weakened, and a larger proportion of the guided energy is converted into radiated waves, leading to improved radiation efficiency. The ripple-like fluctuations in both curves above 14 GHz arise from the frequency-dependent nature of the leaky-wave radiation mechanism. At certain frequencies, the guided wave experiences stronger leakage, which suppresses inter-element coupling and enhances radiation efficiency. At other frequencies, the leakage rate is lower, allowing residual guided energy to propagate between elements, leading to higher S21 values and corresponding dips in efficiency. These periodic variations confirm that the trade-off between radiation and inter-element coupling is frequency-dependent and directly influences the array’s total efficiency.

Table 2 compares the proposed flexible conformal leaky-wave antenna (LWA) with several previously reported LWA designs. Besides the operating bandwidth, scanning angle range, peak gain, and dielectric substrate type, we also include the beam-scanning coverage so that the frequency-scanning performance can be compared more directly. As the table shows, some reported designs achieve a comparable peak gain or endfire radiation, but they usually rely on rigid substrates such as FR4, Rogers 5880, and F4B, and therefore lack mechanical flexibility and conformal adaptability. Moreover, their operating bandwidths are considerably narrower, and their beam-scanning ranges are more limited than those of the proposed design. By contrast, the proposed LWA adopts a PI-ABS composite flexible substrate, which enables stable radiation performance under curved conditions while maintaining a compact conformal configuration. It achieves a wide operating bandwidth of 6 GHz (from 12 to 18 GHz), an ultra-wide beam-scanning range from −67° to 32°, and a peak gain of 16.5 dBi. Overall, the proposed design provides a reasonable balance among wide bandwidth, broad scanning coverage, high gain, and structural flexibility, which is difficult to achieve with conventional rigid LWAs.

## 5. Conclusions

This paper proposes a conformal LWA array based on a PI-ABS composite flexible substrate structure. The array is composed of four flexible LWAs, and each LWA is designed with periodic metallic branches that cooperate with tapered radiating patches to achieve synergistic radiation, thus successfully realizing a flexible conformal array with continuous beam-scanning capability. Simulation and experimental measurement results demonstrate that the proposed conformal array can adapt well to curved substrates while retaining the flexibility of the individual antenna. By virtue of the array configuration, the antenna array exhibits improved engineering characteristics such as enhanced directional radiation and concentrated energy radiation. This approach provides a feasible solution for the design and implementation of flexible conformal directional radiation systems in the microwave band (Ku-band), and it holds great application prospects in the field of satellite communications. Future work will extend the proposed array to a larger-scale configuration, where independent amplitude and phase control at each feeding port enables more flexible beam steering. Meanwhile, fabrication precision and assembly consistency will be improved by adopting laser-assisted alignment, vacuum lamination to eliminate air gaps between the PI film and the ABS substrate, and soldered SMA connectors for stable contact. In addition, we will place emphasis on the measurement of full multi-port S-parameters in future research to achieve a more accurate evaluation of inter-array coupling characteristics.

## Figures and Tables

**Table 1:** Proposed unit dimensions (mm).

l_1_	w_1_	l_2_	w_2_	l_3_	w_3_	l_4_	w_4_	l_5_	w_5_	l_6_	w_6_	l_7_	w_7_	l_8_	w_8_	l_9_	w_9_
291	38	2.1	0.1	0.2	2.1	1.95	0.2	2	5.9	2.05	2	243	14	5.1	1	4.3	2
l_10_	w_10_	l_11_	w_11_	l_12_	w_12_	l_13_	w_13_	l_14_	w_14_	l_15_	w_15_	l_16_	w_16_	l_17_	t	h_1_	h_2_
4.1	1	3.3	2	3.1	1	2.3	2	2.1	1	1.3	2	1.1	1	0.3	0.017	0.1	1

**Table 2:** Comparison with other LWAs.

Works	Operating Bandwidth (GHz)	Bandwidth (GHz)	Scanning Angle	Maximum Gain (dB)	Dielectric Substrate/Radome of Antenna
[9]	9.25–10.34	1.09	endfire	16.32	FR4
[1]	5.6–6.1	1	−46–53°	6.6	F4BTM320 + Rogers 5880
[10]	16.25–17	0.75	−27–33°	8.32	Rogers 5880
[23]	5.38–5.75	0.37	endfire	11.6	F4B
Our work	12–18	6	−67–32°	16.5	PI + ABS

## Data Availability

The original contributions presented in the study are included in the article, further inquiries can be directed to the corresponding authors.

## References

[1] Zheng W., Wang J., Zhao H., Li Z., Geng Y., Li Y., Chen M., Zhang Z. (2023). A leaky wave antenna with capability of fixed-frequency beamforming scanning. IEEE Trans. Antennas Propag..

[2] Fu Q., Liu L., Luo G.Q. (2023). A flexible millimeter-wave endfire antenna based on spoof surface plasmon polaritons. IEEE Antennas Wirel. Propag. Lett..

[3] He Z., Lin X., Yang X., Li C., Xm Y. (2023). A flexible microwave ablation antenna for lung cancer treatment. IEEE Antennas Wirel. Propag. Lett..

[4] Tang H., Bulger C.J., Rovere T., Zheng B., An S., Li H., Dong Y., Haerinia M., Fowler C., Gonya S. (2022). A low-profile flexible dual-band antenna with quasi-isotropic radiation patterns for MIMO system on UAVs. IEEE Antennas Wirel. Propag. Lett..

[5] Gan L., Huang H., Liu X., Yu S., Jing X., Cui Y. (2025). A Low-Profile Flexible Tri-band Antenna for Both Communication and Navigation Uses on AAVs. IEEE Antennas Wirel. Propag. Lett..

[6] Lee H.J. (2025). Cylindrical Polydimethylsiloxane (cPDMS)-Resonator Antenna for Broadband and Flexible Radio-Frequency Electronic Devices. IEEE Antennas Wirel. Propag. Lett..

[7] Samal P.B., Chen S.J., Fumeaux C. (2023). Flexible hybrid-substrate dual-band dual-mode wearable antenna. IEEE Trans. Antennas Propag..

[8] Zhang G., Tian J., Zhang X., Liu J., Lu C. (2022). A flexible planarized biconical antenna for partial discharge detection in gas-insulated switchgear. IEEE Antennas Wirel. Propag. Lett..

[9] Wang S., Hou Y., Teng X., You C., Yuan Y., Yan N., Luo Y., Ma K. (2025). A High Gain Periodic Leaky-Wave Conformal Endfire Antenna Array Based on HISL Technology for X-Band Applications. IEEE Antennas Wirel. Propag. Lett..

[10] Nagaraju D., Verma Y.K. (2021). A compact conformal stub-loaded long slot leaky-wave antenna with wide beamwidth. IEEE Antennas Wirel. Propag. Lett..

[11] Zhu W., Song W., Hu J., Guo Z., Wang L., Yi Z. (2025). Phase-Variable and Matching-Tunable Smart Antenna Using Flexible PDMS Cavity for Adaptive Wireless Communications. IEEE Trans. Antennas Propag..

[12] Wang S., Wang W., Zheng Y. (2025). Dual-functional quasi-uniform beam-scanning antenna array with endfire radiation capability for integrated sensing and communication applications. IEEE Trans. Veh. Technol..

[13] Li M., Chen S.L., Liu Y., Guo Y.J. (2023). Wide-angle beam scanning phased array antennas: A review. IEEE Open J. Antennas Propag..

[14] Sarkar A., Pham D.A., Lim S. (2022). 60 GHz electronically tunable leaky-wave antenna based on annular surface plasmon polariton media for continuous azimuth scanning. IEEE Trans. Antennas Propag..

[15] Mu H., Ding C., Meng F., Zhang Y., Wang J. (2025). Cosinusoidal Phase Modulation Jamming Using Tunable Metasurface Against SAR-GMTI. IEEE Trans. Antennas Propag..

[16] Dang Q.H., Chen S.J., Nguyen-Trong N., Fumeaux C. (2023). Dual-band frequency-reconfigurable wearable antennas for UHF radio band applications. IEEE Trans. Antennas Propag..

[17] Mutai K.K., Chen Q. (2024). Compact leaky wave fed frequency-scanning gradient index lens antenna for near-field-focusing applications. IEEE Trans. Antennas Propag..

[18] Vadher P., Sacco G., Nikolayev D. (2023). Meandering microstrip leaky wave antenna with dual-band linear–circular polarization and suppressed open stopband. IEEE Trans. Antennas Propag..

[19] Ding C., Mu H., Shi Y., Wu Z., Fu X., Zhu R., Cai T., Meng F., Wang J. (2025). Dual-polarized and Conformal Time-Modulated Metasurface Based Two-Dimensional Jamming Against SAR Imaging System. IEEE Trans. Antennas Propag..

[20] Ding C., Mu H., Meng Y., Zhao M., Zhang Y., Cai T., Meng F., Wang J. (2025). Time-Modulated Metasurface-Assisted Moving Target Jamming for Synthetic Aperture Radar. IEEE Trans. Microw. Theory Tech..

[21] Li M., Zhang Z., Tang M.C., Yi D., Ziolkowski R.W. (2020). Compact series-fed microstrip patch arrays excited with Dolph–Chebyshev distributions realized with slow wave transmission line feed networks. IEEE Trans. Antennas Propag..

[22] Song L.Z., Qin P.Y., Guo Y.J. (2020). A high-efficiency conformal transmitarray antenna employing dual-layer ultrathin Huygens element. IEEE Trans. Antennas Propag..

[23] Duan J., Zhu L. (2021). A compact narrow-width endfire leaky wave antenna on coplanar stripline. IEEE Trans. Antennas Propag..

[24] Eddin F.B.K., Fen Y.W., Cui K., Liew J.Y.C., Lim H.N., Fauzi N.I.M., Daniyal W.M.E.M.M., Hou S. (2025). Performance Analysis of Plasmonic Sensor Modified with Chitosan-Graphene Quantum Dots Based Bilayer Thin Film Structure for Real-Time Detection of Dopamine. Prog. Electromagn. Res..

[25] Liao D., Wang C., Zhu X., Jing L., Li M., Wang Z. (2025). Global Designed Angle-Multiplexed Metasurface for Holographic Imaging Enabled by the Diffractive Neural Network. Prog. Electromagn. Res..

